# Assessment of Trimetazidine Treatment in Acute Myocardial Infarction Patients Undergoing Percutaneous Coronary Intervention

**DOI:** 10.1155/2022/7674366

**Published:** 2022-07-02

**Authors:** Xiuying Tang, Jie Gong

**Affiliations:** ^1^Department of Cardiology, The First Hospital of QinHuangDao, QinHuangDao 066000, HeBei, China; ^2^Department of Cardiology, Hebei Medical University, ShiJiaZhuang 050000, HeBei, China

## Abstract

**Aims:**

Trimetazidine (TMZ) is effective at improving clinical outcomes in chronic heart failure and stable coronary artery disease patients. However, no single study has comprehensively evaluated the efficacy of TMZ in acute myocardial infarction (AMI) patients undergoing percutaneous coronary intervention (PCI).

**Methods:**

We enrolled 401 Chinese patients. All patients received the same drug prescription except for TMZ. In blinded fashion, patients were randomized to either a control or an experimental group in which 60 mg TMZ was provided at admission and then at 20 mg three times a day thereafter. At 2 and/or 6 days, we evaluated creatine kinase (CK and CK-MB), cardiac troponin I (cTnI), C-reaction protein (CRP), serum tumor necrosis factor (TNF-*α*), serum creatinine (Cr), serum urea, glucose, glutamic pyruvic transaminase (ALT), and glutamic oxaloacetic transaminase (AST). Additionally, by echocardiography, we assessed left ventricular ejection fraction (LVEF), left ventricular end-diastolic dimension (LVEDD), and cardiac output (CO).

**Results:**

CK and CKMB, which were recorded on the second day in the hospital (each *p*=0.022), and cTNI, which was recorded on the sixth day in the hospital (*p*=0.003), were reduced with TMZ treatment compared to control. In addition, ALT and AST (*p*=0.001, *p*=0.000, respectively) and glucose after 6 days (*p*=0.011) were significantly lower in the study group than in the control group. Furthermore, LVEF after 10–14 days and 6 months after discharge (*p*=0.039 and *p*=0.047, respectively) was increased with TMZ treatment. The effects of TMZ on CRP, TNF-*α*, Cr, urea, LVEDD, and CO were not significant (all *p* > 0.05).

**Conclusions:**

For AMI patients undergoing PCI, TMZ reduced circulating biomarkers of myocardial infarction, reduced values of ALT, AST, and glucose, and improved cardiac function compared with the control group.

## 1. Introduction

Acute myocardial infarction (AMI) may be accompanied by hyperglycemia, hepatic insufficiency, and renal failure [1–4]. Despite substantial progress in pharmacotherapeutic and invasive treatments [5–7] in recent decades, successful treatment of AMI and the disturbances of the endocrine system accompanied by AMI remains elusive for many patients.

Because of changes in human life rhythm and diet, the incidence of hyperglycemia and acute myocardial infarction is increasing. A sedentary lifestyle and poor cardiorespiratory fitness are independent risk factors for coronary atherosclerotic heart disease [8]. Conversely, proper exercise and good cardiorespiratory fitness are very important not only for AMI patients but also for healthy people [9, 10]. Hyperglycemia is one of the main determinants of adverse outcomes in AMI patients. It is common for stress hyperglycemia to occur followed by AMI for nondiabetic patients. Previous studies have shown that stress hyperglycemia followed by AMI is positively associated with adverse outcomes, including morbidity and mortality, especially among elderly patients without diabetes [11–13]. In addition, hyperglycemia has a significant negative impact on restenosis for acute ST-segment elevation myocardial infarction (STEMI) that underwent primary percutaneous coronary intervention (PPCI) [13]. Treatment and monitoring of hyperglycemia are necessary for the prognosis of AMI patients. Trimetazidine (TMZ) is a cytoprotective drug that modifies cardiac muscle metabolism, to provide anti-ischemic benefits. TMZ inhibits *β*-oxidation of free fatty acids (FFA) and then shifts FFA to the more efficient glucose oxidation and thus helps to preserve the ATP level in myocardial cells and result in a positive effect. Through this mechanism, TMZ is thought to correct disturbances in myocardial cellular homeostasis that result from acute ischemic damage and to protect against cardiomyocyte injury [14, 15]. In this regard, TMZ may protect cardiomyocytes against excessive oxygen free radical (OFR) production that occurs in ischemia and inflammation and that initiates oxidation of lipids and proteins and damages DNA.

Previous studies showed that exercise time and left ventricular ejection fraction were significantly increased; cardiac function and clinical symptoms were improved; the area of myocardial infarction was reduced; and all-cause mortality, hospitalization, and major adverse cardiac events (MACEs) over 12 months were decreased with TMZ [15–17]. The efficacy of TMZ in stable coronary artery disease, chronic heart failure, and even AMI has been well-demonstrated. However, to date, no comprehensive evaluations of the efficacy of TMZ have been performed in AMI patients undergoing PCI and its subsequent metabolic disturbances. Therefore, we performed this current study to evaluate the efficacy of TMZ in such a study population and to not only evaluate relevant metabolic parameters including glucose metabolism but also observe inflammatory biomarkers.

## 2. Methods

### 2.1. Patient Population

We enrolled patients diagnosed with AMI at the First Hospital of Qinhuangdao, China, between January 1, 2018, and January 1, 2021.

The inclusion criteria were as follows: (1) age >18 years; (2) diagnosed with AMI in accordance with the guidelines for the management of acute myocardial infarction; (3) underwent PCI; (4) Killip class ≤3; and (5) the provision of informed consent.

The exclusion criteria were as follows: (1) cardiogenic shock; (2) life-threatening diseases or malignant tumor; (3) contraindications to TMZ; (4) severe hepatic and/or renal function failure; (5) serious anemia which was defined as haemoglobin less than 60 g/L; (6) no provision of informed consent; (7) suspected mechanical complications of AMI (septal rupture, wall rupture, or ischemic mitral valve regurgitation); and (8) development of CABG after angiography.

AMI was defined according to the guidelines of the European Society of Cardiology [18]. The local ethics committee, which is affiliated with the First Hospital of Qinhuangdao, approved the study (no. 201501A005).

### 2.2. Drug Strategy

All patients received the same drug regimen except for TMZ. We used a random number table to randomize patients into a control group or to an experimental group for whom a loading dose of 60 mg TMZ was provided at admission [19, 20] and then provided three times a day for at least 14 days thereafter. All patients received percutaneous coronary intervention (PCI) treatments. All researchers involved in this study were physicians.

Absent contraindications: all patients received conventional drugs including ACEIs/ARBs, *β*-blockers, and statins in accordance with the guidelines for the management of acute myocardial infarction [18, 21, 22].

### 2.3. Study Endpoints

We studied the effect of TMZ on cardiac troponin I (cTnI), serum creatine kinase and its isoenzyme (CK and CK-MB), serum urea, serum creatinine (Cr), serum glutamic pyruvic transaminase (ALT), serum glutamic oxaloacetictransaminase (AST), blood glucose, left ventricular end-diastolic dimension (LVEDD), C-reaction protein (CRP), serum tumor necrosis factor (TNF-*α*), left ventricular ejection fraction (LVEF), and cardiac output (CO).

### 2.4. Follow-Up

Data were collected on admission, except for echocardiography results. CK, CKMB, cTnI, CRP, and TNF-*α* were retested at 6 AM on the second day after admission. All data collection was performed at 6 AM six days after the initiation of treatments, which took place 1 day before discharge, except for CK, CKMB, CRP, TNF-*α,* and the echocardiography index. In addition, echocardiography was performed by GE Vivid E95 again 10 to 14 days after initial hospitalization and 6 months after discharge.

### 2.5. Sample Calculation and Statistical Analysis

We used the Sample Size Calculations in Clinical Research tool to calculate the primary endpoints. A previous study [16] determined that compared with control treatments, myocardial injury markers were significantly reduced by TMZ: cTnI was 0.384 ± 0.527 in the TMZ treatment group and 0.631 ± 0.472 in the control group. In our study, a greater improvement was expected in the TMZ group. For a significance level of 5% and power of 80%, we need to include more than 40 patients per group to evaluate the benefits and side effects of TMZ. We need to include 40 patients per group to evaluate the benefits and side effects of TMZ. But we included more than 40 patients per group, where possible, because of the number of hospitalized patients, lost to follow-up, a study design incorporating multiple outcome variables, and exclusion criteria. We included 430 total patients, with 215 each in the control and TMZ groups.

We use SPSS v20.0 (SPSS Inc., Chicago, IL) to perform all data analyses. Student's *t*-test was used to analyze continuous variables expressed as mean ± SD. The chi-square was used to analyze the dichotomous variables. All comparisons were two-tailed, and *p* < 0.05 was considered statistically significant.

## 3. Results

### 3.1. Patient Characteristics

**Figure 1:**
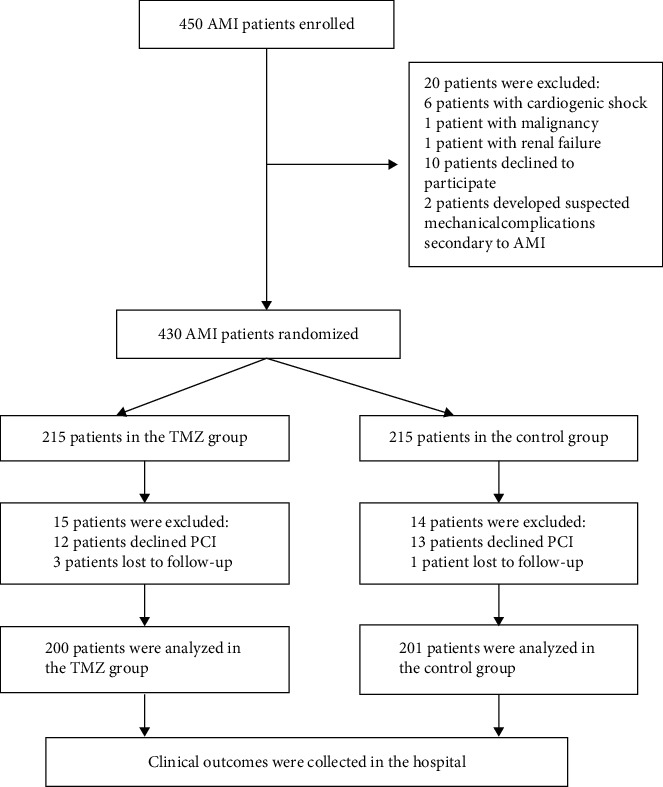
Flowchart of patient enrollment. PCI, primary percutaneous coronary intervention; AMI, acute myocardial infarction; TMZ, trimetazidine.

We included 430 AMI patients undergoing PCI. Fifteen patients were excluded from the TMZ group: twelve declined PCI and three were lost to follow-up after leaving the hospital. Fourteen patients were excluded from the control group: thirteen declined PCI; and one was lost to follow-up. 29 patients were excluded because of lost to follow-up. Patient data were collected as shown in Figure 1 (Figure 1).

Four hundred and one patients completed this study: 200 in the TMZ treatment group and 201 in the control group. The baseline characteristics of the patients are presented in Tables 1 and 2. 72% were males, 49% had hypertension, and 43% had diabetes. The proportion of included patients that had TIMI grade 0–2 flow before PPCI was not different between groups. Routine treatments were prescribed to all patients (Table 2). Baseline biochemical criteria (ALT, AST, Glu, Cr, and urea) and myocardial injury markers were similar (Table 3).

### 3.2. Study Endpoints

The TMZ treatment had no effect on cTNI levels on day two in the hospital (*p*=0.092), but after six days in the hospital, cTNI (*p*=0.003) levels were significantly lower in the TMZ group compared with the control group (Table 4). However, compared to the control group, CK and CKMB were significantly reduced in the TMZ treatment group (*p*=0.022). CRP and TNF-*α* were not obviously improved in both the TMZ treatment group and the control group (*p*=0.554 and *p*=0.968, respectively). LVEDD and CO were not significantly improved in the TMZ-treated patients, including both inpatients and outpatients (LVEDD: *p*=0.371, *p*=0.482; CO: *p*=0.402, and *p*=0.701, respectively). However, TMZ statistically increased LVEF (Simpson method: *p*=0.039 and *p*=0.047, respectively), albeit modestly, after 10 to 14 days and 6 months after discharge (Table 5). TMZ did not significantly improve renal function, as evidenced by Cr and urea (*p*=0.583 and *p*=0.843, respectively), but TMZ appeared to significantly improve ALT and AST after 6 days of treatment (*p*=0.001 and *p*=0.000, respectively). Glucose was lower in the treatment group after 6 days (*p*=0.011) (Table 4).

### 3.3. Sensitivity Analysis

The efficacy of TMZ in patients who were treated with primary PCI—a more homogenous population—was conducted using sensitivity analyses. In these patients, LVEDD, CO, LVEF, Cr, and urea were not significantly different between groups (*p*=0.339, *p*=0.395, *p*=0.082, *p*=0.267, and *p*=0.742, respectively). The data revealed that CK, CKMB, TNI, ALT, AST, and glucose were significantly reduced compared with the control group (*p*=0.001, *p*=0.001, *p*=0.018, *p*=0.001, *p*=0.000, and *p*=0.019, respectively). These data are shown in Tables 6 and 7.

## 4. Discussion

In our study, the effect of TMZ was evaluated in AMI patients undergoing PCI. Moving beyond prior work, we evaluated effects on subsequent parameters. A previous study [23] demonstrated that the BNP levels were decreased, LVEDD was reduced, and myocardial performance index (MPI) was increased. A previous study showed that the serum levels of myocardial enzymes and coronary heart disease severity were positively correlated [24]. Thus, the current study examined myocardial enzymes as an indicator of the amount of infarcted myocardium. Though our study did not demonstrate a significant effect on LVEDD, CO, and cTNI on the second day in the hospital, our study did show that TMZ statistically increased LVEF modestly after ten to fourteen days in the hospital and 6 months after discharge, decreased the amount of infarcted myocardium (as evidenced by the CK and CKMB on the second day and of cTNI after 6 days of treatment), and improved heart function. The above effects of TMZ on myocardial injury biomarkers are consistent with the effects of TMZ in primary PCI patients. It is reasonably likely that such patients would recover faster, have reduced hospital stays, and incur lower expenditures. These results are similar to those reported in acute coronary syndrome (ACS) patients [14–17]. cTNI on the second day, LVEDD, and CO were similar between the two groups, but these potentially may result from a type 2 error given our small sample. A high-quality randomized controlled trial with larger sample size and longer follow-up should be performed to clarify these results as well as evaluate the cost-effectiveness of TMZ.

Acute myocardial infarction (AMI) is a common cardiovascular disease that causes tissue damage, triggers a series of inflammatory processes, and even results in death [25]. Events, such as endothelial injury, that can lead to a hyperactive and pr-inflammatory phenotype of endothelial cells, first cause coronary atherosclerotic heart disease [25]. CRP and TNF-*α* are some early markers of inflammation and tissue damage [26–30]. CRP has been shown to be predictive of early and future cardiovascular events, and TNF-*α* is related to myocardium structure and left ventricular remodeling [26–30]. Therefore, our study used CPR and TNF-*α* as study parameters. Previous studies showed that certain biomarkers of inflammation are elevated during AMI, especially CRP and TNF-*α*, and the levels of CRP and TNF-*α* may determine the degree of myocardial damage and prognosis of heart disease [26–30]. In our study, there were no significant differences from the control group with respect to CRP and TNF-*α* after the second day of TMZ treatment. We speculate that this is because of the pharmacokinetics of the TMZ tablets, which involves a significant lag time between oral intake and effect.

Hyperglycemia, hepatic insufficiency, and renal failure may result from the lower perfusion pressures in the setting of AMI-induced heart failure [1–4]. These high-risk factors complicating AMI showed a clear association with morbidity, mortality, and medical prognosis, and the identification of these events may guide interventions to improve outcomes for AMI patients [31]. Statins are recommended in patients with AMI, unless there are contraindications, according to the guidelines for the management of acute myocardial infarction [18, 21, 22]. Proprotein convertase subtilisin/kexin type 9 (PCSK9) antibodies were added to statins for reducing LDL-C when statins could not decrease LDL-C effectively [32, 33]. However, statins may have adverse effects on skeletal muscle [34] and renal function and cause increases in liver enzymes susceptibility to type 2 diabetes mellitus [35, 36]. Hyperglycemia after AMI can increase morbidity, mortality, and in-stent restenosis [11–13]. Hepatic dysfunction may be accompanied by higher all-cause mortality and bleeding [37]. In our study, TMZ appears to significantly reduce ALT and AST and moved glucose levels toward normal after 6 days of TMZ treatment. Although there are other possible explanations for the improved values, TMZ was beneficial for AMI patients, especially for those with dysmetabolic syndrome. We speculate that TMZ may ameliorate some adverse effects of statins on metabolic disturbances.

A previous study showed impaired renal function and anemia to be independent risk factors for mortality in AMI patients [38]. A renal function served as an endpoint in this study. Unfortunately, unlike in a previous trial [39], TMZ did not appear to improve renal function as assessed by Cr and urea.

In contrast with the reported study, the effects of TMZ in primary PCI showed that LVEDD, CO, LVEF, Cr, and urea were not different between groups. This may result from the small sample size. However, we found that TMZ may significantly reduce CK, CKMB, cTNI, ALT, AST, and modulate glucose in AMI patients treated with primary PCI.

TMZ clearly had substantial effects on blood markers such as CK, CKMB, cTNI, ALT, AST, and blood glucose, and these indicators of TMZ are consistent with our previous trial [40]. However, its effect on LVEF appears modest (although not zero) in terms of its clinical relevance.

### 4.1. Study Limitations

Our study was modest in size for a cardiac intervention study, although based on our available data which was sufficiently well-powered to demonstrate clinically meaningful changes in the outcome variables evaluated, even if it might miss clinically irrelevant smaller effects. We had a short follow-up of 6 months. ACE inhibitors and beta-blockers were used in less than 50% of the included patients usually because of low blood pressure or lower heart rate. In such settings, therapy with nitrates was preferred. The use of ACE inhibitors and beta-blockers between groups was not significantly different. Larger, high-quality randomized controlled trials with longer follow-up periods and more homogeneity will be helpful to verify our findings, particularly those findings that are inconsistent with prior data. The peak levels of cardiac injury biomarkers were not compared between groups; so, the myocardial infarct size was not accurately assessed. Physical activity is beneficial to cardiac antioxidant capacity and the elasticity of coronary arteries [41], and the total exercise time was not assessed in AMI patients after TMZ treatments with a longer follow-up, for example, at six months. For the duration of the total period, we will pay attention to the assessment of physical activity in future clinical trials. Finally, the short- and long-term mortality was not evaluated.

## 5. Conclusions

TMZ may reduce circulating biomarkers of myocardial infarction, reduce values of ALT, AST, and blood glucose, and improve LVEF vs. the control group in AMI patients undergoing PCI.

## Figures and Tables

**Table 1:** Baseline clinical characteristics.

Indices	TMZ group (*n* = 200)	Control group (*n* = 201)	*p* value
Age (years)	64.3 ± 11.3	64.2 ± 10.9	0.877
Male sex (%)	143 (72)	146 (73)	0.800
Hypertension (%)	95 (48)	103 (51)	0.453
Diabetes (%)	89 (45)	84 (42)	0.584
Dyslipidemia(%)	87 (44)	72 (36)	0.116
Smoker (%)	113 (57)	126 (63)	0.207
Prior MI (%)	25 (13)	16 (8)	0.134
Angina pectoris (%)	104 (52)	109 (54)	0.655
Peripheral arterial disease (%)	13 (7)	17 (8)	0.456
Atrial fibrillation/flutter	10 (5)	15 (7)	0.308
Prior stroke/TIA (%)	30 (15)	32 (16)	0.799
Killip (%)			
1	108 (54)	103 (51)	0.580
2	77 (39)	78 (39)	0.950
3	15 (8)	20 (10)	0.385
Culprit lesion (%)			
LAD	102 (51)	116 (58)	0.177
LCX	44 (22)	36 (18)	0.306
RCA	54 (27)	49 (24)	0.548
Number of stents	1.2 ± 0.4	1.2 ± 0.4	0.691
PPCI	145 (73)	135 (67)	0.244
TIMI grade 0-2 before PCI (%)	144 (72)	133 (66)	0.207
Manual thrombus aspiration (%)	17 (9)	19 (9)	0.739

MI, myocardial infarction; TIA, transient ischemic attack; PPCI, primary percutaneous coronary intervention; TIMI, thrombolysis in myocardial infarction.

**Table 2:** Pharmacologic treatments.

	TMZ group (*n* = 200)	Control group (*n* = 201)	*p* value
Aspirin	200	201	—
Ticagrelor	200	201	—
Anticoagulant (LMWH)	200	201	—
*β*-Blockers	92	98	0.580
ACEIs/ARBs	78	91	0.203
Statins	198	199	1.000
Nitrates	142	151	0.352

LMWH, low-molecular-weight heparin calium; ACEI, angiotensin-converting enzyme inhibitor; ARB, angiotensin receptor blocker.

**Table 3:** Baseline laboratory data at admission.

	TMZ group (*n* = 200)	Control group (*n* = 201)	*p* value
CK (U/L)	214 ± 152	203 ± 117	0.446
CKMB (U/L)	28 ± 15	26 ± 13	0.238
cTNI (ng/ml)	1.9 ± 1.8	1.6 ± 1.6	0.117
ALT (U/L)	37.2- ± 23.4	36.8 ± 22.3	0.888
AST (U/L)	117.3 ± 132.9	112.4 ± 97.6	0.670
Cr (*μ*mol/L)	81.8 ± 24.6	79.1 ± 28.9	0.316
Urea (mmol/L)	7.1 ± 1.9	6.9 ± 8.4	0.789
Glucose (mmol/L)	7.8 ± 2.9	7.6 ± 2.8	0.363

CK and CK-MB, serum creatine kinase and its isoenzyme; cTNI, cardiac troponin I; Cr, serum creatinine; ALT, serum glutamic pyruvic transaminase; AST, serum glutamic oxaloacetic transaminase.

**Table 4:** Repeated laboratory data collected in the hospital.

	TMZ group (*n* = 200)	Control group (*n* = 201)	*p* value
CK on day 2 (U/L)	838 ± 732	1046 ± 1048	0.022^*∗*^
CKMB on day 2 (U/L)	86 ± 71	106 ± 104	0.022^*∗*^
CRP on day 2 (pg/ml)	101.5 ± 193.5	121.1 ± 225.9	0.554
TNF-*α* on day 2 (pg/ml)	51.3 ± 83.4	51.8 ± 87.4	0.968
cTNI on day 2 (ng/ml)	14.3 ± 14.5	17.0 ± 17.9	0.092
cTNI on day 6 (ng/ml)	3.5 ± 3.5	4.7- ± 4.9	0.003^*∗*^
ALT on day 6 (U/L)	33.8 ± 25.0	43.6 ± 32.7	0.001^*∗*^
AST on day 6 (U/L)	42.1 ± 26.8	62.8 ± 54.8	<0.001^*∗*^
Cr on day 6 (*μ*mo l/L)	80.7 ± 26.3	82.2 ± 28.0	0.583
Urea on day 6 (mmol/L)	6.3 ± 4.8	6.5 ± 11.6	0.843
Glucose on day 6 (mmol/L)	6.0 ± 1.7	6.4 ± 1.9	0.011^*∗*^

All comparisons were two-tailed, and ^*∗*^*p* < 0.05 was considered statistically significant.

**Table 5:** Inhospital (10–14 days) and postdischarge (6 months) echocardiograms indexes ($\bar{x}$ ± s).

	TMZ group (*n* = 200)	Control group (*n* = 201)	*p* value
LVEDD (mm)	52.3 ± 5.2	52.8 ± 4.7	0.371
	53.5 ± 4.9	53.2 ± 4.6	0.482
CO (L/min)	5.5 ± 1.0	5.4 ± 0.8	0.402
	5.3 ± 0.8	5.3 ± 0.7	0.701
LVEF (%)	56.7 ± 9.3	54.9 ± 7.9	0.039^*∗*^
	54.7 ± 8.3	56.2 ± 7.0	0.047^*∗*^

LVEF, left ventricular ejection fraction; LVEDD, left ventricular end-diastolic dimension; CO, cardiac minute output. All comparisons were two-tailed, and *∗p* < 0.05 was considered statistically significant.

**Table 6:** Repeated laboratory data collected in the hospital.

	TMZ group (*n* = 145)	Control group (*n* = 135)	*p* value
CK on day 2 (U/L)	1012 ± 683	1380 ± 1123	0.001^*∗*^
CKMB on day 2 (U/L)	101 ± 65	138 ± 113	0.001^*∗*^
cTNI on day 2 (ng/ml)	18.7 ± 14.6	23.4 ± 18.3	0.018^*∗*^
ALT on day 6 (U/L)	35.3 ± 27.9	48.0 ± 35.9	0.001^*∗*^
AST on day 6 (U/L)	42.8 ± 29.4	67.0 ± 61.5	<0.001^*∗*^
Cr on day 6 (*µ*mo l/L)	77.4 ± 17.2	80.2 ± 24.7	0.267
Urea on day 6 (mmol/L)	6.2 ± 5.3	6.6 ± 14.0	0.742
Glucose on day 6 (mmol/L)	5.9 ± 1.4	6.3 ± 1.5	0.019^*∗*^

All comparisons were two-tailed, and *∗p* < 0.05 was considered statistically significant.

**Table 7:** Inhospital (10–14 days) echocardiograms indexes ($\bar{x}$ ± s).

	TMZ group (*n* = 145)	Control group (*n* = 135)	*p* value
LVEDD (mm)	52.1 ± 5.0	52.6 ± 4.6	0.339
CO (L/min)	5.5 ± 1.0	5.4 ± 0.9	0.395
LVEF (%)	56.5 ± 9.1	54.7 ± 8.0	0.082

LVEF, left ventricular ejection fraction; LVEDD, left ventricular end-diastolic dimension; CO, cardiac minute output.

## Data Availability

The datasets generated during and/or analyzed during the current study are not publicly available, but are available from the corresponding author on reasonable request.
